# Job-search self-efficacy and reemployment willingness among older adults: roles of achievement motivation and age

**DOI:** 10.1186/s12877-021-02645-5

**Published:** 2021-12-07

**Authors:** Shen Liu, Zijing Hong, Wei Zhou, Yingfen Fang, Lin Zhang

**Affiliations:** 1 Department and Institute of Psychology, Ningbo University, Ningbo, 315211 China; 2 School of Humanities and Social Sciences, University of Science and Technology of China, Hefei, 230022 China; 3 School of Management, Jinan University, Jinan, 511486 China

**Keywords:** Older adults, Reemployment willingness, Job search self-efficacy, Achievement motivation, Age

## Abstract

**Background:**

The present study aimed to explore the relationship between job-search self-efficacy and reemployment willingness among older adults, as well as roles of achievement motivation and age in this relationship.

**Methods:**

Three hundred and sixty-five Chinese retired older adults were recruited from five neighborhoods in a city via convenience sampling, and they were measured by the Job Search Self-Efficacy Scale and the Achievement Motivation Scale (AMS).

**Results:**

Results revealed that job-search self-efficacy significantly positively predicted reemployment willingness. Achievement motivation played a partial mediating role in the relationship between the job-search self-efficacy and reemployment willingness. Age moderated the relationship between job-search self-efficacy and reemployment willingness.

**Conclusions:**

These findings showed that increasing the job-search self-efficacy and achievement motivation could effectively promote older adults’ reemployment willingness. The present study provided a theoretical basis for caring for older adults’ reemployment willingness.

## Introduction

Reemployment of older adults can not only promote the development of the labor resources, but also change the consumer pension into a productive pension, which not only alleviate the pressure of social endowment, but also effectively protect such individuals’ physical and mental health [18, 36]. Reemployment of older adults has become an important way in China for social engagement. In China, the retirement age of Chinese workers is 60 for men and 50 for women. In order to resist the pressure of population aging, Chinese government takes measures to promulgate the implementation principle of “delayed retirement” in 2021,^1^ which creates a policy protection environment for older adults’ reemployment. However, the individual’s willingness to reemployment will also be restricted by physical and psychological factors [10]. Some studies have found that factors such as the health, age, education level, and economic status have a significant impact on older adults’ reemployment willingness [7]. Therefore, this study bases on older adults’ mental state, and aims to explore the internal mechanism between job-search self-efficacy and older adults’ reemployment willingness to help alleviate the pressure of social endowment, and realize the benefits of older adults.

Bandura [5] believed that, since different tasks require different skills and abilities, individuals’ self-efficacy levels can vary across different task fields. In the job-search field, job-search self-efficacy refers to the ability and confidence of individuals to complete job searching [23]. When people has a high level of job-search self-efficacy, they will have better expectations of job-search behaviors, and accompanied by more positive emotions [31]. This study focused on the reemployment willingness of older adults before reemployment behavior, which refers to the willingness of the older adults over 60 years old to reemployment [7]. Theory of planned behavior, which suggests that a person’s attitude toward a certain behavior, their subjective norms, and the perceived degree of difficulty controlling and performing the behavior can determine his/her intentions towards that behavior [21]. Job-search self-efficacy represents a positive attitude towards job-seeking intentions, which can motivate individuals to complete job-seeking behaviors. It could be speculated that older adults’ cognitive evaluation and perceived behavioral control regarding job-search behaviors may determine their level of job-search self-efficacy. Studies of the reemployment of young workers who had lost their jobs indicated that job-search self-efficacy significantly influenced reemployment willingness [6, 9, 20, 31].. Consequently, we proposed:

### *Hypothesis 1:* job-search self-efficacy would positively predict the reemployment willingness of older adults

Motivation plays a guiding and sustaining role in decision-making and individual behavior [22]. Achievement motivation is the internal motivation and psychological tendency of striving for excellence to achieve higher goals, which can be divided into two motivational tendencies: pursuing success and avoiding failure [1]. Maehr and Kleiber [16] believed that achievement motivation may change from an external and competitive achievement model to an internal mastery related model with the growth of age, and they may pay more attention to short-term goals than long-term goals. Studies conducted among young people have reported that achievement motivation can significantly predict an individual’s job-search intention [29]. The reemployment behavior of the older adults can maintain the social role of the older adults, help them maintain a stable life in their old age, effectively maintain mental health [33]. Therefore, it can be speculated that, among older adults, achievement motivation significantly predicts older adults’ reemployment willingness. In addition, studies have found a significant correlation between self-efficacy and achievement motivation [26]. Self-efficacy theory posits that the motivation that drives an individual’s behavior constitutes cognitive activity that is focused on responding to a certain situation; this is closely related to self-efficacy [3]. In other words, when searching for jobs, older adults perform cognitive evaluations of their own abilities and the difficulty of the job-search task. Based on this, we can speculate that older adults with high job-search self-efficacy, compared with those with low job-search self-efficacy, have higher cognitive evaluations of their own abilities, higher levels of achievement motivation, and higher reemployment willingness. Based on this, we proposed:

### *Hypothesis 2:* achievement motivation could play a mediating role in the relationship between the job-search self-efficacy and reemployment willingness of older adults

Reemployment willingness is restricted by many factors, such as gender, age, and education level. Of these, age has a significant effect on the reemployment willingness of older adults; that is, the reemployment willingness of older adults weakens with increasing age [19]. A possible reason for this is that age moderates the process by which achievement motivation affects individuals’ psychological and behavioral activities [11, 21]. Studies have shown that the achievement motivation of older adults is significantly lower than that of younger individuals [15]. Age-related factors (such as the decline of health level) will reduce the achievement motivation of the older adults [14]. As mentioned, according to self-efficacy theory, the motivation for individual behavior is based on the individual’s inner cognitive activity when considering performing the behavior, and the strength of motivation depends on the evaluation result of completing a certain task. It is speculated that with the increase of age, older adults may evaluate task difficulty and their own abilities more negatively, creating lower achievement motivation. Consequently, we propose:

### *Hypothesis 3:* age could play a moderating role in the first half of the mediating path of “job-search self-efficacy → achievement motivation → reemployment willingness”

The existing research showed that age had a significant impact on achievement motivation, and there was a significant difference between the achievement motivation of older adults and young adults. Also, the reemployment willingness had differences between older adults who was in 60–69 years old, 70–79 years old and over 80 years old. With the increase of age, the reemployment intention decreased [19]. According to the Losing Age Concept, as individuals’ age, they experience a loss of ability in many aspects [35]. Their general psychological changes show a declining trend, as do their physical, psychological, and activity capacity [15]. Further, as age increases, older adults’ attitudes and methods of acting focus more on their surrounding environments, gradually showing an obvious trend from active to passive, and from the outside world to the inner world [30]. By avoiding work, retired older adults generally pay less attention to the outside world, and lose their sense of competition and need for achievement; therefore, older age tends to predict lower willingness to work [8]. This study concludes that, in the process of reemployment of older adults, the role of achievement motivation in predicting the reemployment willingness of such individuals diminishes with age. Consequently, we proposed:

### *Hypothesis 4:* age would play a moderating role in the second half of the mediating path of “job-search self-efficacy → achievement motivation → reemployment willingness”

Above-mentioned studies have found that job-search self-efficacy significantly impacts reemployment willingness. However, these studies generally targeted certain age groups, and ignored the effect of age on individuals. The current study intends to verify the possible moderating role of age in the relationship between job-search self-efficacy and reemployment willingness among older adults. By focusing on the reemployment willingness of older adults at different ages, the current study infers that reemployment willingness differs among older adults of different ages and those who have different levels of job-search self-efficacy. In particular, old older adults have more severe physical decline, and their reemployment willingness is more susceptible to psychological factors; that is, job-search self-efficacy. Consequently, we proposed:

### *Hypothesis 5:* age could play a moderating role in the direct path by which job-search self-efficacy affects the reemployment willingness of older adults

**Fig. 1:**
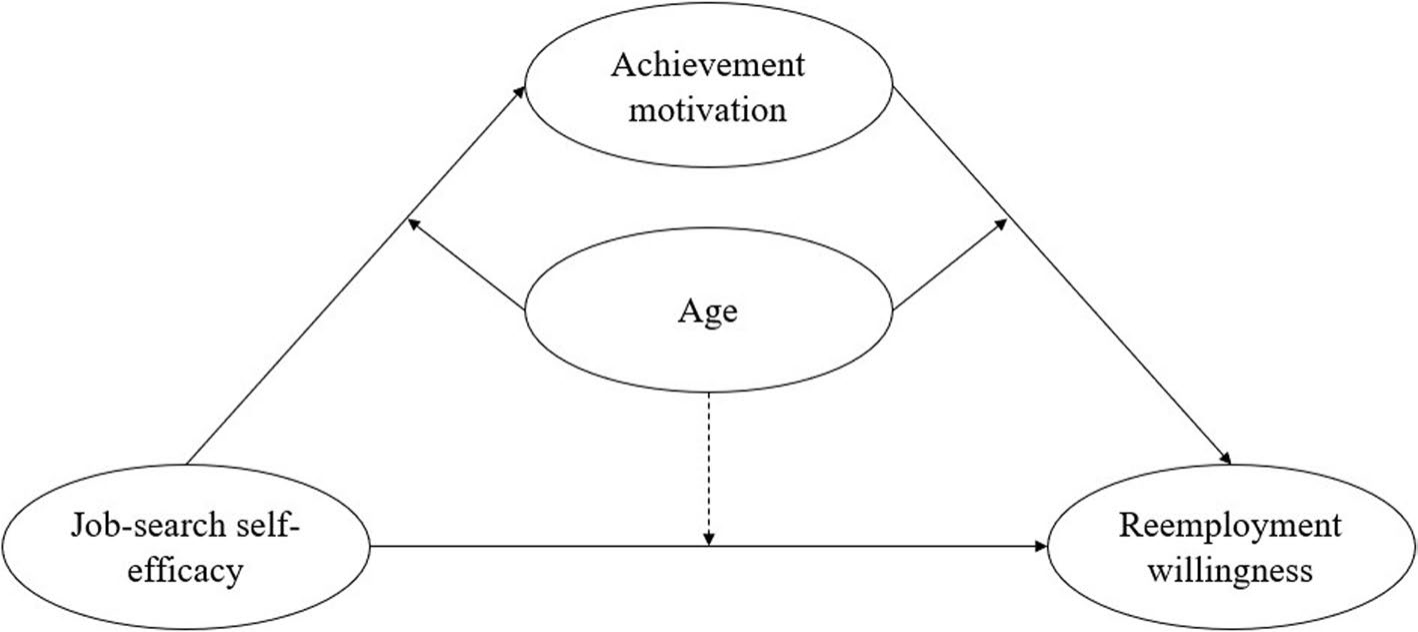
Moderated mediation model

In summary, the current study explores the relationship between job-search self-efficacy and reemployment willingness among older adults. Specifically, the following aspects are investigated: (1) Whether achievement motivation plays a mediating role in the relationship between the job-search self-efficacy and reemployment willingness of older adults. And (2) whether age moderates the mediating process by which job-search self-efficacy affects the reemployment willingness of older adults. The hypothetical model of the relationship between the variables is shown in Fig. 1.

## Methods

### Participants

Three hundred and sixty-five Chinese retired older adults were recruited from five neighborhoods in a city via convenience sampling. The inclusion criteria in this study were retired older adults aged 60 years and above who did not have severe physical disability or weakness, and who had normal cognition and the ability to answer questions. The older adults have jobs before retirement, including enterprise employees, government officials, education, entertainment, medical and health, etc. Consequently, 358 completed questionnaires were collected and analyzed, giving an effective response rate of 98.08%. The full sample (i.e., the original 365) comprised 194 males and 171 females. They were aged between 60 and 89 years, with an average age of 70.93 years (*SD* = 7.94). Regarding education level, 130 respondents had primary education or lower, 117 had junior high school education, 69 had high school education, and 42 had college education or higher. For monthly income, 116 respondents received less than 2000 RMB, 74 received between 2000 and 3000 RMB, 79 received between 3000 and 5000 RMB, and 89 received over 5000 RMB. After the test, the participants received certain rewards or equivalent gifts. The Ethics Committee of authors’ University approved this study, in accordance with the ethical principles of the Declaration of Helsinki. Participants were given informed consent and it was written. The ethics committee also formally approved this consent.

### Materials

#### Job-search self-efficacy

The Job Search Self-Efficacy Scale, compiled by Van et al. [25] and revised by Song et al. [21], was adopted to measure the participants’ job-search self-efficacy. This is an eight-item questionnaire, such as “make the best impression and express your opinion in the interview”, for which items are scored using a five-point Likert-type scale ranging from 1 (“not sure”) to 5 (“very sure”). The score is the sum of all items, the higher the score, the higher the job-search self-efficacy level. For the current study, Cronbach’s α coefficient for the scale was 0.89.

#### Achievement motivation

The Achievement Motivation Scale (AMS), compiled by and Gjesme and Nygard [17] and revised by Ye [32], was adopted to measure the participants’ achievement motivation. This is a 30-item questionnaire (such as “I like work that I can do with my best”), and the items are scored using a four-point Likert-type scale ranging from 1 (“completely inconsistent”) to 4 (“completely consistent”). AMS includes two dimensions: motivation to pursue success and motivation to avoid failure. The final score is calculated by subtracting the score for motivation to pursue success from the score for motivation to avoid failure. The higher the score, the stronger the achievement motivation. In the current study, the Cronbach’s α coefficients for the scale and the two dimensions were 0.88, 0.71, and 0.86, respectively.

#### Reemployment willingness

Referring to the methods of Vinokur et al. [24] and Chen et al. [7], the current study used one question (Would you like to look for a job in the next month?) to measure the participants’ reemployment willingness. To respond to this question, a five-point Likert-type scale was used, ranging from 1 (“very unwilling”) to 5 (“very willing”); the higher the score, the stronger the reemployment willingness.

### Measurement procedure

In order to conduct a collective test, a community approach was applied. Well-trained undergraduate students majoring in psychology served as the experimenters. The participants answered the questionnaire independently, after confirming that they had understood the requirements. The participants with high education levels answered the questionnaire after receiving instructions from the experimenters; meanwhile, those with low education levels answered the questionnaire after receiving detailed explanations from the experimenters. All of the participants answered the questionnaire without interference. Questionnaires were distributed and collected in the same session. After excluding invalid questionnaires (those who missed filling, did not answer the question seriously, and withdrew halfway), SPSS 22.0 was used to conduct stepwise regression. Model 1 and Model 4 in the PROCESS macro program (download address: http://www.afhayes.com/download; [12]) were used to test the specific moderating and mediating effects. Meanwhile, AMOS 24.0 was used to test the integrated model.

## Results

### Common method Bias test

Since all data represented subjective reports from participants, there was a risk that the results would be affected by common method bias. Consequently, following the approach of Zhou and Long [34], the Harman single factor test was used to test the deviation of the common method. The results showed that 10 factors had eigenvalues greater than 1. The variation amount explained by the first factor was 21.09%, far less than the critical value of 40%, indicating that there was no obvious common method deviation in the current study.

### Means, standard deviations, and correlation matrices of the variables

The means, standard deviations, and correlation matrices of the variables in the current study are shown in Table 1. The participants’ education levels were significantly positively correlated with their monthly income and reemployment willingness; education level was significantly negatively correlated with achievement motivation; job-search self-efficacy and achievement motivation were significantly positively correlated with reemployment willingness; age was significantly negatively correlated with reemployment willingness; and job-search self-efficacy was significantly positively correlated with achievement motivation. Thus, gender, education level, and monthly income were considered control variables for subsequent analysis.

**Table 1:** Means, Standard Deviations, and Correlation Matrices of the Study Variables (*n =* 359)

	*M*	*SD*	1	2	3	4	5	6	7
1 Gender^a^	–	–	1						
2 Education level^b^	–	–	−0.01	1					
3 Monthly income^c^	–	–	−0.01	0.71^***^	1				
4 Age	70.93	7.54	−0.36^***^	−0.08	0.04	1			
5 Job-search self-efficacy	20.09	6.19	−0.01	0.11^*^	0.04	−0.02	1		
6 Achievement motivation	3.25	6.54	−0.03	−0.11^*^	−0.11^*^	0.04	0.20^***^	1	
7 Reemployment willingness	2.89	1.12	0.07	0.75	−0.16	−0.45^***^	0.18^***^	0.37^***^	1

*Note*

^a^is a virtual variable, 0 = male, 1 = female

^b^is a virtual variable, 1 = below primary, 2 = primary school, 3 = junior high school, 4 = high school, 5 = college

^c^is a virtual variable, 1 = less than 2000, 2 = 2000–3000, 3 = 3000–5000, 4 = over 5000. *n* = 358

^*^p < .05

^**^p < .01

^***^p < .001

### Moderated mediation model test

Based on the moderated mediation test proposed by Wen and Ye [27], the current study explored the relationship between the participants’ job-search self-efficacy and reemployment willingness. The mediating effect of achievement motivation and the moderating effect of age in the above relationship were also investigated. After normalizing all variables, three regression equations were tested through stepwise analysis. Equation 1 predicted the moderating effect of age on the relationship between the participants’ job-search self-efficacy and reemployment willingness; equation 2 predicted the moderating effect of age on the participants’ job-search self-efficacy and achievement motivation; and equation 3 predicted the moderating effect of age on the participants’ achievement motivation and reemployment willingness. The test results are shown in Table 2. Equation 1 showed that job-search self-efficacy positively predicted reemployment willingness among the participants (*p* < 0.001), that age negatively predicted their reemployment willingness (*p* < 0.01), and that the interaction between job-search self-efficacy and age significantly predicted their reemployment willingness. Meanwhile, equation 2 showed that job-search self-efficacy had a significant predictive effect on achievement motivation (*p* < 0.001). Finally, the results for equation 3 indicated that job-search self-efficacy had a significant predictive effect on the participants’ reemployment willingness (*p* < 0.01), and that achievement motivation and age also significantly predicted their reemployment willingness (*p* < 0.001); moreover, the interaction between job-search self-efficacy and age significantly predicted their reemployment willingness (*p* < 0.05). In summary, after analyzing the moderated mediation effect, it was found that achievement motivation played a partial mediating role in the relationship between the participants’ job-search self-efficacy and reemployment willingness, while age played a moderating role in the direct path of their job-search self-efficacy and reemployment willingness. Consistent with our Hypothesis 1, 2, 5, moderated mediation test revealed that job-search self-efficacy was positively related to reemployment willingness via achievement motivation, and age moderated the relationship between job-search self-efficacy and reemployment willingness. Hypothesis 3 and Hypothesis 4 of this study were not supported.

**Table 2:** Analysis of the Moderated Mediation Effect

	Equation 1 (validity criterion: reemployment willingness)			Equation 2 (validity criterion: achievement motivation)			Equation 3 (validity criterion: reemployment willingness)		
	*β*	*SE*	95%CI	*β*	*SE*	95%CI	*β*	*SE*	95%CI
Job-search self-efficacy	0.65^***^	0.47	[0.13,0.33]	0.45^***^	0.51	[0.10,0.30]	0.15^**^	0.05	[0.07, 0.24]
Age	−0.73^***^	0.17	[−0.85, − 0.39]	0.17	0.19	[−0.19, 0.54]	−0.42^***^	0.05	[−0.52, − 0.32]
Job-search self-efficacy × age	0.92^*^	0.49	[0.01,0.92]	−0.38	0.54	[−0.44, 0.67]	1.09^*^	0.45	[0.20,0.98]
Achievement motivation × age							0.09	0.45	[−0.79, 0.97]
Achievement motivation							0.36^***^	0.05	[0.26, 0.45]
Gender	−0.11^*^	0.11	[− 0.43, − 0.01]	−0.02	0.12	[−0.27, 0.19]	−0.10^*^	0.10	[−0.40, − 0.02]
Education level	0.04	0.06	[−0.08, 0.15]	−0.09	0.06	[−0.20, 0.05]	0.07	0.06	[−0.04, 0.16]
Monthly income	−0.05	0.06	[−0.15, 0.07]	−0.06	0.06	[−0.17, 0.07]	−0.03	0.06	[−0.13, 0.08]
*R*^2^	0.23			0.04			0.34		
*F*	17.55^***^			3.85^**^			23.29^***^		

*Note.* Each variable in the model was normalized

At the same time (see Table 3), when age was low (average minus a standard deviation), the mediating effect value of achievement motivation was 0.04, and the lower limit of Bootstrap 95% confidence interval was − 0.09, while the upper limit was 0.16. Zero was contained, which indicated not significant results. When age was high (average plus a standard deviation), the mediating effect value of achievement motivation was 0.24, and the lower limit of Bootstrap 95% confidence interval was 0.12, while the upper limit was 0.36. Zero was not contained, which indicates significant results. The above results showed that the mediating effect of achievement motivation only existed at high levels of age.

**Table 3:** The mediating role of achievement motivation under different levels of age

Mediating variable	Age	Indirect effect value	Boot standard error	Boot CI lower limit	Boot CI upper limit
Achievement motivation	*M-SD*	0.04	0.06	−0.09	0.16
	*M*	0.14	0.04	0.05	0.23
	*M + SD*	0.24	0.06	0.12	0.36

*Note*: Boot Standard Error, Boot CI Lower Limit, Boot CI Upper Limit refers to the standard error of indirect effect estimated by percentage method with deviation correction, the lower and upper limit of 95% confidence interval, and all values are rounded to reserve two decimal numbers

**Fig. 2:**
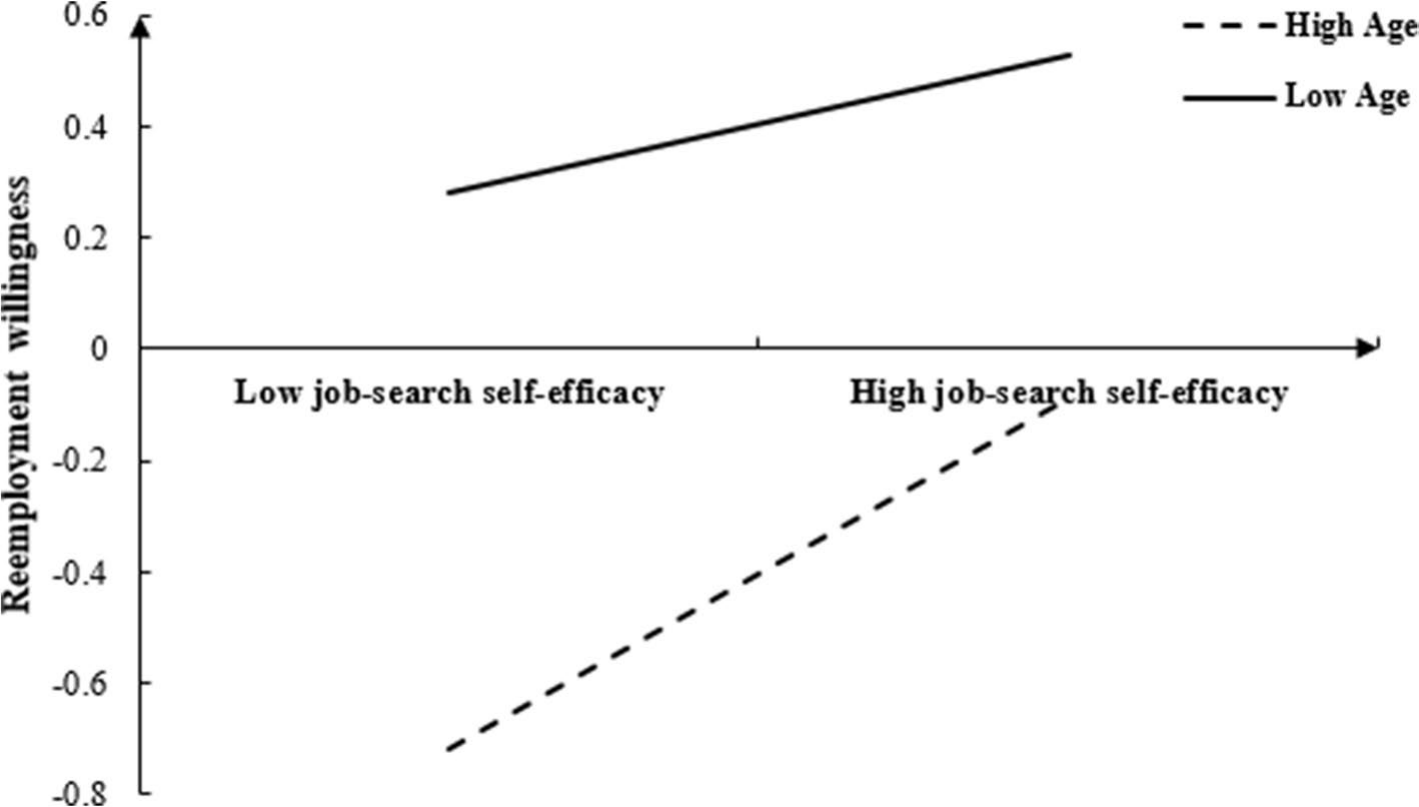
Moderating effect of age

In order to better explain the moderated mediating model, age was divided into two groups based on plus or minus one standard deviations. A simple slope test was used to investigate the effect of job-search self-efficacy on reemployment at different age levels (see Fig. 2). As shown in Fig. 2, for younger participants the predictive effect of job-search self-efficacy on their reemployment willingness was not significant (*β* = 0.12, *t* = 1.80, *p* = 0.070); meanwhile, for older participants job-search self-efficacy significantly positively predicted their reemployment willingness (*β* = 0.32, *t* = 4.72, *p* < 0.001). This shows that as age increases, job-search self-efficacy develops a stronger predictive effect on reemployment willingness.

**Fig. 3:**
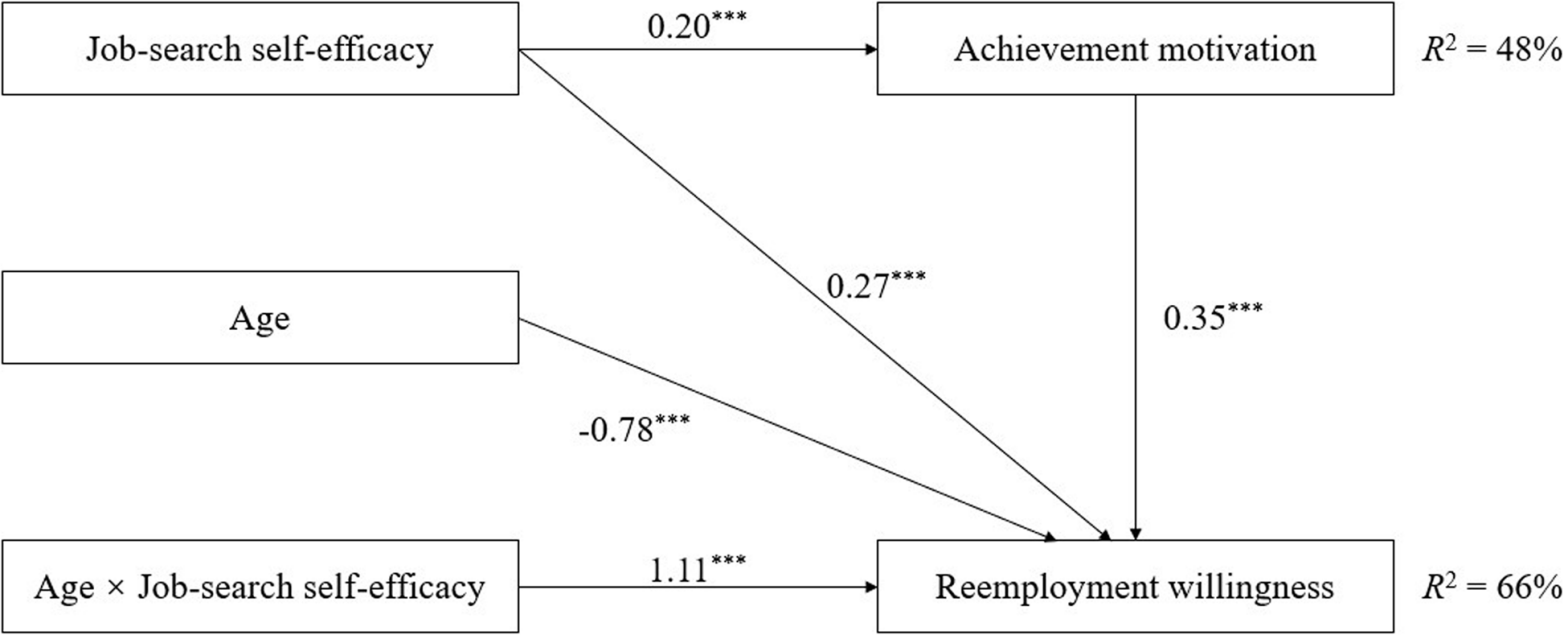
Moderated mediation model. Note. *R*^2^ indicates variance explanation

The integration model was further tested by applying the suggestions of Wen et al. [28], the results showed that the model fitted well (*χ*^2^/*df* = 2.32, CFI = 0.91, NFI = 0.90, GFI = 0.93, RMSEA = 0.07; see Fig. 3). Job-search self-efficacy significantly positively predicted the reemployment willingness of the older adults (γ = 0.27, *p* < 0.001), and significantly positively predicted achievement motivation (γ = 0.20, *p* < 0.001), while achievement motivation also significantly positively predicted reemployment willingness (γ = 0.35, *p* < 0.001). These findings indicate that achievement motivation plays a partial mediating role in the relationship between job-search self-efficacy and reemployment willingness among older adults. Age significantly negatively predicted reemployment willingness (γ = − 0.78, *p* < 0.001); in contrast, the interaction between age and job-search self-efficacy significantly positively predicted reemployment willingness (γ = 1.11, *p* < 0.001). These findings indicate that age has a moderating effect on the direct path mediated by job-search self-efficacy.

## Discussion

The present study took China as the research background, and retired older adults as the research participants. It has found that job-search self-efficacy significantly positively predicts the reemployment willingness of older adults; this finding conforms to the self-efficacy theory and the theory of planned behavior. Job-search self-efficacy has an important influence on individuals’ job-search intention and behavior, while behavioral intention is the most direct factor affecting behavior. Since self-efficacy and perceived behavioral control are conceptually interlinked, the findings support the effect of perceived behavioral control on behavioral intention in the theory of planned behavior. Therefore, job-search self-efficacy affects individuals’ job-search behaviors by influencing their job-search intentions [5]. The results of the present study generally proved that job-search self-efficacy affects the reemployment willingness of older adults; that is, the higher the level of job-search self-efficacy, the stronger the reemployment willingness. Level of job-search self-efficacy reflects older adults’ confidence and cognition of their reemployment competence, and directly affects their tendency to engage in this psychological and behavioral activity (in other words, their reemployment willingness).

### Mediating effect of achievement motivation

This indicates that job-search self-efficacy affects the reemployment of older adults through achievement motivation. The size of the direct effect and indirect effects are 0.15 and 0.07. The results of the current study conform to the concept of the self-efficacy mechanism in human agency [4]. In particular, the present findings show that the primary effect of job-search self-efficacy occurs through the mediation of achievement motivation. More specifically, previous studies have shown that, like younger individuals, achievement motivation significantly predicts the reemployment willingness of older adults [29]. According to the continuum theory of aging proposed by Atchley [2], the older adults try to maintain a consistent life style before and after retirement, so as to reduce the undesirable interference. One way for the older adults to achieve continuity is to reemployment [13]. In addition, the older adults will face many psychological crises, such as the transition from working all day to a clean and comfortable life after retirement, and the transformation from work unit centered to family centered interpersonal relationship. These changes are all problems that every older adults should take seriously. With the growth of age, the older adults will face various problems brought about by aging, and their understanding of achievement motivation is also more inclined to avoid failure. Some studies have found that the score of failure avoidance dimension in achievement motivation of the older adults recognition group is higher than that of the young group [15]. Reemployment of the older adults helps to overcome the disappointment caused by the changes of daily life and interpersonal relationship brought about by retirement, and can obtain a certain source of income to meet the sense of achievement of the older adults and relieve the economic pressure. Although young people and old people have different understanding of achievement motivation, achievement motivation of the older adults can still predict their reemployment intention. Individuals’ job-search self-efficacy, namely, assessment competence, affects their behavioral motivation, and then affects their behavioral intention. The older adults have a high sense of self-efficacy in the field of job-hunting, and think that they can be competent for the reemployment task. This positive cognitive evaluation is conducive to the formation of high-level achievement motivation. The older adults believe that through reemployment, they can better face the challenges brought by aging and avoid experiencing disappointment and failure in life, which will improve their reemployment willingness. Improving job-search self-efficacy and achievement motivation contributes to increasing the reemployment willingness of older adults. Therefore, society should seek to create a relatively relaxing employment environment that helps older adults increase their job-search self-efficacy. After retirement, older adults should seek to maintain their work skills as well as good physical and mental health, as this will help them better cope with the challenges of reemployment and, thus, maintain high achievement motivation. Such measures would help older adults become reemployed while also enjoying a high quality of life and level of mental health.

### Moderating effect of age

This study found that age plays a moderating role in the relationship between job-search self-efficacy and the reemployment willingness of older adults. The results also show that age has a negative predictive effect on the reemployment willingness of older adults, which is consistent with the findings of previous studies [19]. Meanwhile, age was found to enhance the predictive effect of high job-search self-efficacy on the reemployment willingness of older adults. Job-search self-efficacy showed an insignificant predictive effect on the reemployment willingness of young older adults, but its predictive effect on reemployment willingness was found to increase with age. However, the reemployment willingness of the oldest participants was generally much lower than that of the younger participants, and it remains at a relatively low level; this finding is consistent with those of previous studies [8, 19]. For older adults, as age increases, physical function and work skills decrease significantly. Previous research has found that the age of reemployed older adults tends to concentrate on the early stage of old age (below 70 years); such individuals tend to have maintained good physical condition and to have accumulated more social and life experience [15]. This explains why young older adults generally maintain a high level of reemployment willingness, and are less affected by job-search self-efficacy. However, old older adults show a relatively lower level of reemployment willingness, as they have been removed from their employment for a long time and have already adapted to retirement life; further, they also have less knowledge of advanced working skills and modern job-search tools [8]. Moreover, as age increases, older adults gradually shift their attitude from the outside world to the inner world [30]. Thus, the decline of physiological functions and changes in psychology increase the predictive effect of job-search self-efficacy on the reemployment willingness of old older adults. Consequently, young older adults should be the focus of the older-adult reemployment cause in China. The country should expand the information channels through which older adults can seek employment, popularize the concept of working in old age, and strengthen relevant policies for facilitating older adults’ reemployment. Older adults should be encouraged and guided to become reemployed, as this would allow them to make contributions to the development of society, both in spiritual and material terms.

### Significance

By considering the factors of job-search self-efficacy, achievement motivation, and the age of older adults, the current study reveals the internal mechanism of older adults’ reemployment willingness. The results support the concepts advanced by the theory of self-efficacy and the theory of planned behavior. The findings indicate that achievement motivation plays a mediating role in the relationship between job-search self-efficacy and the reemployment willingness of older adults. The results also verify that achievement motivation is an important factor influencing the reemployment intention of older adults. Thus, increasing older adults’ level of job-search self-efficacy and achievement motivation will help increase their reemployment willingness. The current study also revealed how age moderates the impact of job-search self-efficacy on the reemployment willingness of older adults. It is shown that early old age is the most suitable stage for older-adult reemployment. Old older adults generally have less reemployment willingness and are more susceptible to job-search self-efficacy.

The research results can provide references that the government can use to increase the reemployment of older adults. First, the government should fully respect and provide employment opportunities for these older adults who are willing to work, and meet the needs of the older adults for value realization by reducing the idleness of older labor qualifications. Second, the government should help reduce age discrimination in the field of labor and reemployment, and solve the problems of insufficient employment security for the older adults, insufficient recognition or necessary support for the contribution and value of the older adults in the family field, and limited reemployment channels for the older adults. Third, the government should increase re-education and training of older labor to adapt to new changes and work requirements, and maintain a high labor participation rate and production efficiency. Finally, the government should enhance the confidence of the older adults in reemployment, create a good employment environment through cultural propaganda, and provide all aspects of support to maintain their job-search self-efficacy.

### Limitations and future directions

There are limitations to the current study that should be improved in follow-up studies. First, we used a questionnaire survey not a longitudinal research to collect the data, which makes us could not get causal relationship. Second, this study does not control for other factors that affect the reemployment willingness of older adults, such as social security income, health status, the previous career before retirement, the health status, the number of children and the need to take care of their grandchildren [6, 19]. Third, we emphasized the importance and influencing factors of older adults’ reemployment in this study, however, older adults may face more discrimination in this process, increasing research on reducing age discrimination will provide new ideas and programs for the reemployment research. Furthermore, the background of this study only was considered the Chinese context. Due to the inconsistent retirement age and physical and psychological level of older adults in different countries, more cross-cultural research is needed in the future. Finally, this research investigated reemployment willingness, not reemployment behavior, we still need to investigate the consistency of willingness and real behavior.

## Conclusion

The current study obtained the following conclusions: (1) Job-search self-efficacy can significantly positively predict the reemployment willingness of older adults. (2) Achievement motivation can play a partial mediating role in the relationship between job-search self-efficacy and the reemployment willingness of older adults. (3) For older adults, age can moderate the direct path of achievement motivation’s indirect effect on the job-search self-efficacy–reemployment willingness relationship.

## Data Availability

All data used and analyzed in this study are available from the corresponding author on reasonable request.

## Footnotes

1 Source of reference: http://www.gov.cn/
